# Evaluating the impact of the ‘Blow, Breathe, Cough’ health promotion intervention in resolving otitis media with effusion in children: An adaptive randomized-controlled trial protocol

**DOI:** 10.1016/j.conctc.2025.101531

**Published:** 2025-08-07

**Authors:** Jaimee R. Rich, Michael Dymock, Elke J. Seppanen, Elena Montgomery, Tanisha Cayley, Tamara Veselinović, Greta Bernabei, Anri Lester, Amy Hannigan, Nicole Irvine, Kerryn Gidgup, Edna Ninyette, Steph Bray, Tu Trang Tran, Valerie M. Swift, Melinda Edmunds, Natalie Strobel, Daniel McAullay, Julie Marsh, Evelyn Tay, Lea-Ann S. Kirkham, Ruth B. Thornton, Robyn S.M. Choi, Lydia Timms, Emily Jackson, Jafri Kuthubutheen, Peter C. Richmond, Christopher G. Brennan-Jones

**Affiliations:** a Wesfarmers Centre of Vaccines and Infectious Diseases, The Kids Research Institute Australia, Nedlands, Western Australia, Australia; b Western Australian Centre for Rural Health, The University of Western Australia, Geraldton, Western Australia, Australia; c Western Australia Country Health Service, Western Australia, Australia; d School of Allied Health, Faculty of Health Sciences, Curtin University, Bentley, Western Australia, Australia; e School of Population and Global Health, The University of Western Australia, Nedlands, Western Australia, Australia; f Sociology Department, Global Health Program, Princeton University, Princeton, NJ, United States of America; g Perth Children's Hospital, Nedlands, Western Australia, Australia; h Centre for Child Health Research, The University of Western Australia, Nedlands, Western Australia, Australia; i School of Human Sciences, The University of Western Australia, Nedlands, Western Australia, Australia; j Hearing Assessment Program – Early Ears (HAPEE), Hearing Australia, Perth, Western Australia, Australia; k Kurongkurl Katitjin, Edith Cowan University, Mount Lawley, Western Australia, Australia; l School of Medicine, The University of Western Australia, Nedlands, Western Australia, Australia

**Keywords:** Pediatric otolaryngology, Middle ear effusion, Health promotion, Clinical trial protocol, Bayesian models

## Abstract

**Introduction:**

Otitis media with effusion (OME) affects hearing, speech development, and quality of life (QoL) in children. The ‘Blow, Breathe, Cough’ (BBC) intervention promotes nasal, respiratory, and middle ear clearance through nose blowing, deep breathing, coughing, and hand hygiene. It shows promise in resolving OME but lacks randomized-controlled trial (RCT) evaluation. This paper presents a RCT protocol evaluating BBC's effect on OME resolution, hearing, speech, and QoL in children aged two to seven years.

**Methods:**

This parallel-group, 1:1, outcome assessor-blinded, individual adaptive RCT investigates whether completing the BBC intervention plus hand hygiene twice-daily at home increases OME resolution after 4-to-6 weeks in children with OME, compared to hand hygiene alone. Families (n = 250) perform their individually randomized program (BBC plus hand hygiene, or hand hygiene only) at home. The primary outcome is the difference in OME resolution rates between trial arms, assessed using tympanometry (type B to A or C_1_ transition) and otoscopy. Secondary outcomes include natural OME resolution, discharges from tertiary care, family satisfaction, hearing thresholds, QoL, bacterial load in the nasopharynx and on hands, cost comparison within the RCT versus standard care, and adverse events. All outcomes are measured by blinded researchers. An intention-to-treat analysis will be performed on all randomized participants. Guided by an Aboriginal Community Advisory Group, the RCT ensures culturally appropriate research whilst addressing community priorities in managing childhood ear disease.

**Discussion:**

If efficacious, BBC could reform OME treatment, reduce costs, and improve long-term hearing, speech, and QoL outcomes in some children. Its accessibility offers a globally scalable solution.

## Highlights

• Protocol for the inaugural randomized-controlled trial of ‘Blow, Breathe, Cough’.
• Implements an adaptive design, being an innovative approach to clinical trials.
• Primary outcome: difference in proportion of OME resolution between trial arms.
• Potential to reform treatment guidelines for otitis media if proven efficacious.
• Includes an Aboriginal-led Advisory Group to guide culturally appropriate research.

## 1 What is known about this topic?

• Otitis media with effusion (OME) affects children's hearing, speech, and quality of life whilst straining the finances and resources of healthcare systems globally. Cost-effective, non-invasive solutions should be considered to reduce OME prevalence and address delays in otolaryngology care provision for OME.
• Whilst currently recommended for OME prevention in Australia, a study interviewing Australian educators and primary health workers suggested the ‘Blow, Breathe, Cough’ intervention may resolve (treat) OME, although its use in treatment remains unvalidated through clinical trials.

## 2 What does this paper add?

• This paper details the protocol of an adaptive randomized-controlled trial assessing the efficacy of the BBC intervention plus hand hygiene in resolving OME, allowing real-time adjustments to optimize trial efficiency.
• If effective, the BBC intervention plus hand hygiene could reduce the need for surgical intervention in some children and reduce healthcare costs associated with OME, offering a scalable, non-invasive solution accessible across diverse settings globally.

## 3 Introduction

### 3.1 Background and rationale

The human ear, essential for hearing and balance, consists of the outer, middle, and inner ear [1]. The middle ear, including the tympanic membrane, ossicles, and Eustachian tube, facilitates sound transmission to the brain as electrical signals by matching impedance between the air-filled outer ear and fluid-filled inner ear [2]. Otitis media (OM) refers to an array of inflammatory middle ear conditions including otitis media with effusion (OME), where middle ear fluid builds up without acute infective symptoms, disrupting sound conduction and subsequently impairing hearing [3]. In metropolitan Western Australia, OM affects Australian Aboriginal (hereafter respectfully referred to as ‘Aboriginal’) children at a disproportionately higher rate [4]. Whilst 26.8 % of metropolitan Western Australian children are diagnosed with OM by age three years, around 50 % of Aboriginal children experience OM by just six months of age [5,6]. Hearing loss caused by OM can have severe long-term impacts on a child's speech, language, literacy development and behaviour, potentially affecting school readiness [[7], [8], [9]]. It is also associated with reduced school attendance rates for Aboriginal children [10], and is known to negatively influence the educational experience and academic performance of children, which may impact later educational or employment prospects [11]. OM places additional responsibilities, disruptions, and stress on families, with parents voicing particular concerns around its impact on their child's development [12]. As such, OM is a significant risk factor for compromised healthy childhood development, particularly among Aboriginal children [5,6,[13], [14], [15]].

A common treatment option for OME is the prescription of oral antibiotics, particularly for Aboriginal children in remote areas; however, their efficacy may be outweighed by side effects such as diarrhoea, vomiting, and skin rashes, as well as growing concerns around antibiotic resistance [16,17]. The surgical insertion of tympanostomy tubes to aerate the middle ear can improve short-term hearing and reduce OME-related complications; however, they carry risks such as anaesthetic-related issues and tympanic membrane damage, and their long-term benefits remain unclear [14,18,19]. Hearing aids for OME-related hearing loss can also be offered in circumstances where surgical options (such as tympanostomy tubes) are not suitable, are declined, or are ineffective [20]. However, their use remains low amongst Aboriginal children, largely due to the peer-related stigma and negative perceptions associated with hearing aid use [21]. Whilst cost is not always the primary barrier to accessing treatment for OME in Australia, socioeconomic disadvantage experienced by families can significantly impede timely and equitable access to quality otolaryngology care [22]. Contributing factors include challenges navigating the healthcare system, lack of primary care continuity, limited health literacy, and lower uptake of preventative ear health services.

Children requiring tertiary otolaryngology care in Australia endure wait times often exceeding twelve months, with an additional wait of between six and twelve months for surgical treatments such as tympanostomy tube insertion [23]. Annually, treatment costs for all forms of OM are estimated between $100–400 million Australian dollars (AUD) in Australia (approximately $62.8 - $251.3 million United States dollars [USD]), and $5 billion USD in the United States [24,25]. Given OM's substantial impact on childhood development and the strain it imposes on global healthcare systems that precipitates treatment delays, interest is growing in exploring cost-effective, non-invasive solutions to reduce its prevalence and improve outcomes for children with OME [[24], [25], [26], [27]].

The 'Blow, Breathe, Cough’ (BBC) intervention is a simple, freely available, school and family led health promotion intervention encouraging children to blow their nose, breathe deeply, cough, and practice good hand hygiene to assist nasal and respiratory secretion clearance, ultimately encouraging middle ear fluid drainage [28]. Although used anecdotally since the 1970s in Australian classrooms, primary care settings, and Aboriginal community health organizations to prevent ear disease, the BBC intervention plus hand hygiene lacks robust evidence on its efficacy [29]. Findings from interviews with Australian educators and primary healthcare workers suggest the BBC intervention plus hand hygiene may reduce OME and improve hearing in children, decrease the incidence of ear infections, and increase knowledge and awareness of positive hygiene practices [29,30]. Additionally, a non-randomized controlled trial conducted in a school setting demonstrated a statistically significant reduction in signs of upper and lower respiratory tract disease following a daily program of nose blowing, deep breathing, coughing, and exercise among Aboriginal children, although maintaining daily compliance was challenging due to inconsistent school attendance [31]. Considered together, these studies provide preliminary support for evaluating the treatment efficacy of the BBC plus hand hygiene intervention in resolving OME using a randomized-controlled trial (RCT) in a home-based setting. This paper details the protocol for a RCT that will examine the efficacy of the BBC intervention plus hand hygiene in resolving (treating) OME in children in a home-based setting and its impact on hearing, speech, and quality of life (QoL) outcomes.

## 4 Methods

### 4.1 Study design

This parallel group, 1:1, outcome assessor-blinded, individual adaptive RCT asks the following question: *‘Among children aged two to seven years with OME accessing pediatric otolaryngology services, does completing the BBC intervention plus hand hygiene at least twice daily, compared to hand hygiene alone, in a home-based setting increase the proportion of children experiencing OME resolution after four to six weeks?’* This interventional study is a parallel-group, adaptive RCT which employs a two-arm design (the BBC program plus hand hygiene as the intervention, and a hand hygiene only program as the control), featuring a randomization allocation of 1:1 and blinded outcome assessments. As an adaptive RCT, it allows for modifications to procedures based on interim analysis results [32]. The protocol for the RCT is structured in accordance with the Standard Protocol Items: Recommendations for Interventional Trials (SPIRIT) 2013 Statement and RCT results will be reported following the CONSORT 2010 statement for reporting parallel group RCT's [33,34]. This RCT has been registered with the Australian and New Zealand Clinical Trials Registry (ANZCTR) (No. ACTRN12622000546752).

### 4.2 Study setting and sites

Participants are recruited through three pathways: i) children referred to otolaryngology and audiology departments of pediatric hospitals in Western Australia, ii) children referred for surgical intervention for OME (e.g. tympanostomy tube insertion) at pediatric hospitals in Western Australia, and iii) children referred to the Djaalinj Waakinj Centre for Ear and Hearing Health (hereafter referred to as ‘Djaalinj Waakinj’). Recruitment from Western Australian pediatric hospitals will occur primarily at Perth Children's Hospital (PCH) in Nedlands, Western Australia. Meaning ‘listening and talking’ in Aboriginal Noongar language, Djaalinj Waakinj is a service developed with the local Aboriginal community to provide timely diagnosis and intervention for Aboriginal children with ear and hearing issues in southern metropolitan Western Australia [35,36].

### 4.3 Eligibility criteria

Children and their families need to meet all the following inclusion criteria to participate:

1. Aged between two and seven years old.
2. Clinical referral indicates hearing or ear related concerns.
3. Able to attend all study visits regardless of residential location within Western Australia.
4. Persistent OME, defined as two type B tympanometry results documented by a health professional at least three months apart within a 12-month period in the same ear (unilateral or bilateral), with or without hearing loss. These results can be sourced from the child's medical records or collected during screening visits for the RCT. Inclusion of children with persistent OME aligns with the 2020 Otitis Media Guidelines, which recommend intervention when OME persist for three months or longer [4]. To determine eligibility based on persistent OME, children and their families are offered up to three screening visits.

Children and their families are excluded if any of the following exclusion criteria are met:

1. Scheduled to undergo surgical intervention for OME within the next four weeks.
2. Current or previous diagnosis of cleft lip and/or palate, Down syndrome, major chromosomal anomalies, craniofacial anomalies, chronic suppurative otitis media, or congenital ear anomalies.
3. Concurrent participation in another interventional ear study.
4. Unable to engage with study procedures due to any physical, medical, developmental, behavioral or cognitive condition(s).
5. Parent(s)/carer(s) decline to cease using non-study ear clearing interventions during the intervention or control period (any ear treatments that are not clinically prescribed).
6. Clinical inclusion criteria for persistent OME are not met (inclusion criteria no. 4 above).
7. Share a household with a current or previously participating child.

### 4.4 Interventions and comparators

**Fig. 1:**
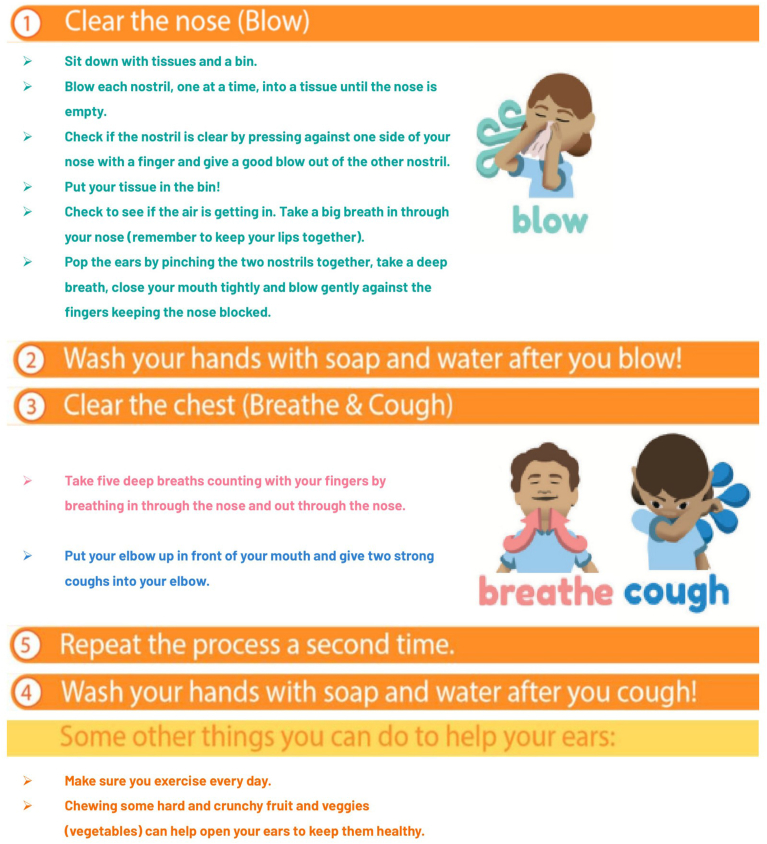
Steps for the ‘Blow, Breathe, Cough’ intervention [28].

**Fig. 2:**
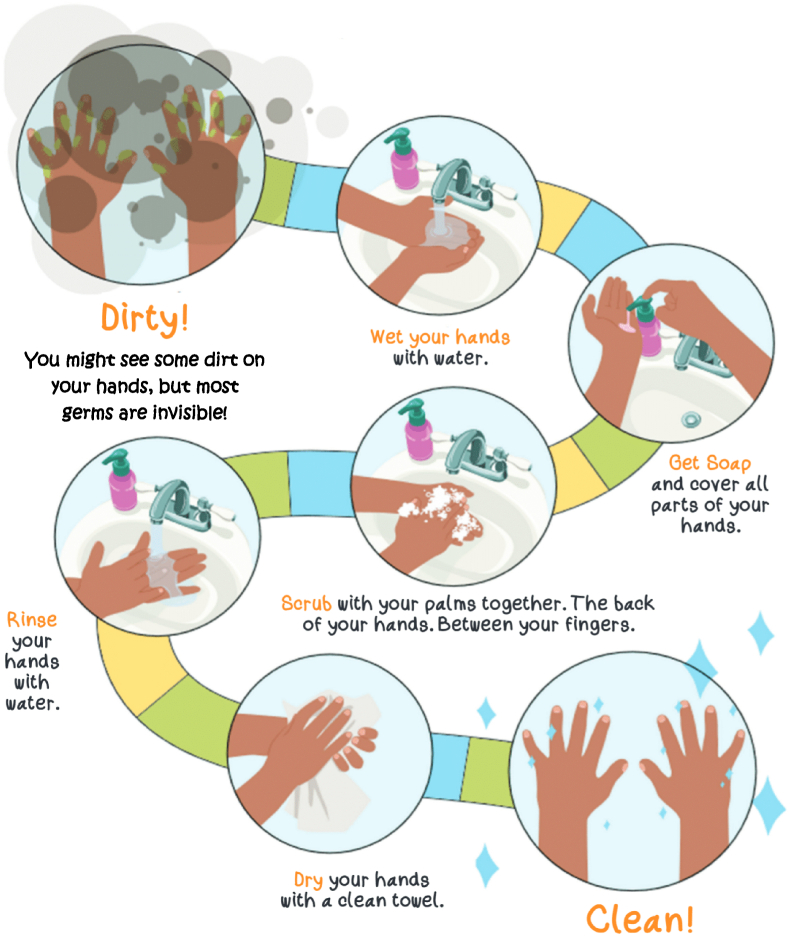
Steps for the hand hygiene program [37].

This study has two arms: the BBC program intervention plus hand hygiene, and a hand hygiene only program control. Families in the intervention arm receive the BBC intervention routine (Fig. 1) to perform at least twice daily for four-to-six weeks, which incorporates the hand hygiene program steps (Fig. 2) [28,37]. The control arm families receive a hand hygiene program in line with Centers for Disease Control and Prevention guidelines, for completion at least twice-daily for four-to-six weeks (Fig. 2) [37]. Trained research staff deliver both programs by conducting interactive demonstrations using step-by-step guides and checklists, and by providing educational materials containing visual prompts to guide program completion. Both arms receive watchful waiting, supportive care, and ear health education (Table 1) [17,38]. Use of ear-specific treatments (e.g. ear candles, EarPopper®, nasal sprays etc.) are prohibited during the four-to-six-week intervention/control period to avoid contamination of the assigned program, with any use of these monitored at each study visit by collecting updated medical histories (Table 2). Adherence is tracked through weekly text message surveys regarding program completion frequency (Table 2). Hand hygiene is recommended by the National Health and Medical Research Council (NHMRC) *Staying Healthy* guidelines to reduce the spread of communicable disease in children, and is embedded within the BBC intervention [39]. It was selected as the control to isolate the effects of blowing the nose, breathing, and coughing on the presence of middle ear fluid whilst engaging control group families in a beneficial, evidence-based hygiene practice [4,29].

**Table 1:** Explanation of additional care provisions within study arms.

**Watchful waiting**	Conducting ear and hearing health assessments at scheduled and as needed time points, with care escalated as clinically indicated.
**Supportive care**	Encouraging parent/carer(s) to raise any questions or concerns about their child's ear health with the research team during study appointments or via phone/text message and to arrange an unscheduled visit as needed or clinically appropriate.
**Generic ear health education resources**	Provision of an information sheet published by the Child and Adolescent Health Service about glue ear (OME).

**Table 2:** Data collection timepoints.

Timepoints	Week 0	Weeks 4–6	Weeks 24–32	Any timepoint
	ScreeningEnrolment	Post-randomization	Final follow-up	Unscheduled visit
Informed consent	•			
Eligibility	•			
Randomization	•			
Medical history	•	•	•	•
Family history	•	**×**	**×**	**×**
Sociodemographic data	•	**×**	**×**	**×**
Otoscopy	•	•	•	•
Tympanometry	•	•	•	•
Audiometry	•	•	•	•
OAE	•	•	•	•
OM-6	•	•	•	•
PLUM	•	•	•	•
HATS	•	•	•	•
Adherence survey (via SMS)	Administered weekly between timepoints			
Intervention program performance checklist	•	•	**×**	**×**
Intervention program parent/carer understanding checklist	•	**×**	**×**	**×**
Motivational outcomes		•	•	**×**
Nasopharyngeal and palm specimen	•	•	**×**	**×**
Adverse event monitoring	Continuous			

• - mandatory; **× -** optional.

### 4.5 Outcomes

The primary outcome measure is the proportion of children that experience OME resolution in the intervention arm compared to the control arm, assessed through tympanometry. Tympanometry is a diagnostic test of middle ear function using parameters such as peak pressure, compliance, and ear canal volume, which tracks the transition from type B result (indicative of OME) at enrolment (week 0) to a type A result (normal) or type C_1_ result (Eustachian tube dysfunction, suggesting resolving OME) at post-randomization (week 4-to-6) (Table 2) [30,40]. For this study, a type A tympanogram is defined as a peak pressure of > −100 daPa, a type B tympanogram has an indeterminable peak pressure, a type C_1_ tympanogram as a peak pressure of between −100 and −199 daPa, and a type C_2_ tympanogram as a peak pressure of −200 to −400 daPa [41]. Type C_1_ tympanograms will be considered indicative of resolved OME in addition to type A tympanograms, as the literature suggests type C_1_ may reflect a transient or resolving stage of middle ear effusion, rather than a persistent form of OME requiring intervention [16,41]. Resolution can be unilateral or bilateral, however the primary outcome will only be considered in any ears that produced type B tympanograms at enrolment. Type C_2_ tympanogram findings will be considered indicative of ongoing middle ear dysfunction. The efficacy of the BBC intervention will be considered clinically meaningful if it results in a number needed to treat to benefit (NNTB) of 10 or less for the resolution of OME.

Secondary outcome measures include: (1) rate of natural OME resolution, evidenced by a transition of type B tympanograms to type A or C tympanograms; (2) proportion of otolaryngology/audiology care discharges, evaluated using multidisciplinary team (MDT) and clinician review outcomes from enrolment to final follow-up (weeks 24-to-32); (3) family satisfaction with care provision through the RCT, assessed using a survey at final follow-up, (4) four-frequency hearing thresholds measured by audiometry at enrolment, post-randomization, and final follow-up; (5) QoL, assessed through the Otitis Media-6 (OM-6), Parent-evaluated Listening and Understanding Measure (PLUM), and Hearing and Talking Scale (HATS) questionnaires at enrolment, post-randomization, and final follow-up; (6) bacterial loads, quantified as the densities of all bacteria species of interest at enrolment and post-randomization, analyzed using high-throughput qPCR on samples collected from the nasopharynx and palms of hands; (7) cost-of-service comparison for OME diagnosis and management through the study compared to standard care via economic analysis; and (8) the proportion of adverse events, monitored through safety data collection from enrolment to final follow-up. Refer to Table 2 for a comprehensive schedule of data collection methods and collection timepoints for participants in both trial arms.

### 4.6 Sample size

Power analysis was used to understand the trial operating characteristics, assuming a range for the proportion of OME resolution in the control arm to be between 14 % and 20 % [16]. Assuming a moderate 13 % absolute increase in OME resolution at post-randomization in the intervention arm compared to the control arm and type one error controlled at 5 %, the power is estimated to be approximately 80 % for a maximum sample size of 250 participants. Accounting for 10 % loss-to-follow-up, the RCT intends to recruit up to 275 participants to achieve sufficient power to declare superiority of the BBC intervention plus hand hygiene.

### 4.7 Recruitment and retention

Participants are recruited from Djaalinj Waakinj and otolaryngology/audiology departments of approved Western Australian pediatric hospital sites (e.g. PCH). Families of children referred to Western Australian hospital sites are informed of the research through an opt-out letter, enabling them to learn of the research participation opportunity whilst preserving their autonomy [42]. Clinicians at Western Australian hospitals may also make direct referrals to the RCT using a consent-to-contact form, which permits research staff to contact families directly about research participation. The information sheet and consent form are sent electronically or by mail to families before their appointment, allowing all relevant family members (e.g. other parents, grandparents, Elders, etc.) to be fully informed about the study, even if they are unable to attend the appointment with the primary parent/carer to provide informed consent. Strategies to promote retention of families recruited from Western Australian pediatric hospital sites include provision of as required support to families via phone, text message, or by reviewing their child at unscheduled time points, flexibility in appointment scheduling to accommodate family needs, and the embedded MDT review process providing families a direct pathway to engage with the hospital system (see full explanation in ‘Clinical oversight’).

Eligible children and families from Djaalinj Waakinj who have provided consent to be contacted for future research are referred to the RCT by Djaalinj Waakinj study clinicians. Both Aboriginal and non-Aboriginal research staff are available to discuss the RCT with families and provide support throughout the informed consent process. Recruitment from the Djaalinj Waakinj pathway enables Aboriginal families to participate in research within a culturally safe, community trusted service [35,36]. The involvement of Aboriginal research staff in study recruitment and conduct, many of whom are well-connected within the local Western Australian south metropolitan community and have long-standing relationships with families participating in research, plays a vital role in upholding culturally safe, respectful research conduct with Aboriginal families [43,44]. Their involvement not only fosters trust and open communication but strengthens the connections between the research team and the community, enhancing participation and retention by ensuring RCT conduct is underpinned by local knowledge and values [43,44].

To acknowledge the time and contribution of families to the research and to support participant retention, enrolled families receive two vouchers intended to contribute towards grocery and fuel expenses. In addition, children will receive a goodie bag containing materials to support the completion of their allocated program (e.g. tissues [BBC only], soap, hand sanitizer, coloring in pages etc.). This approach to participant reimbursement was implemented as recommended by the Aboriginal Community Advisory Group (ACAG).

### 4.8 Allocation-sequence generation, concealment, randomization, and blinding

**Fig. 3:**
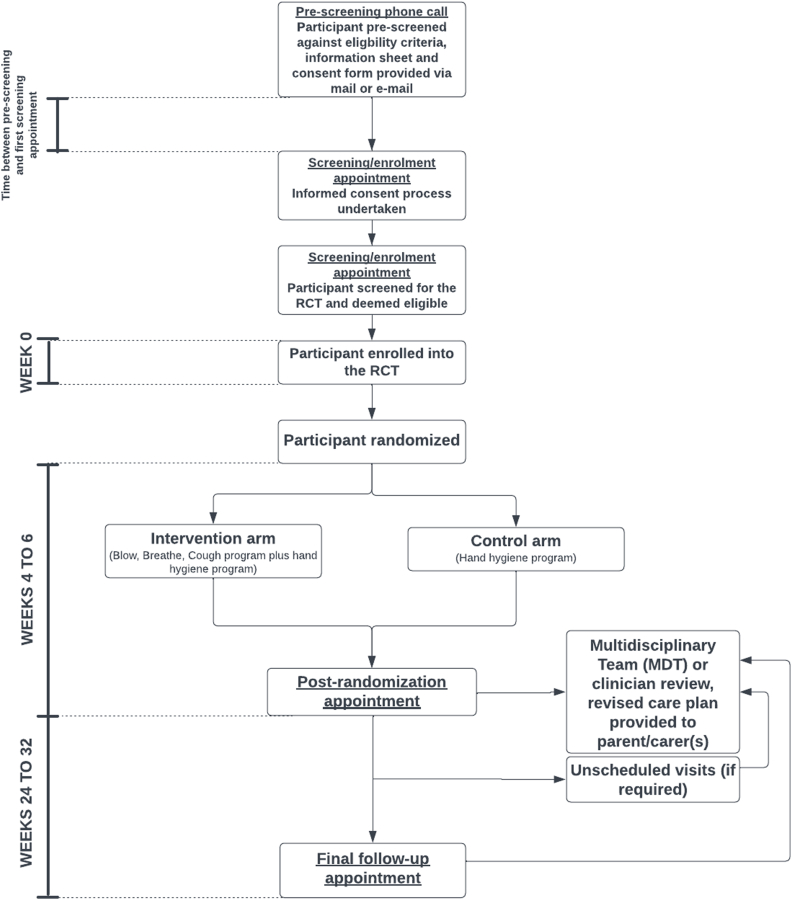
Study flow.

Participants are randomly assigned to the intervention or control arm using a computer-generated list (R v4.1, block sizes 2, 4, 6, 8) [45], stratified by site (PCH, Djaalinj Waakinj, or other Western Australian hospital sites), OME class (unilateral or bilateral), and prior tympanostomy tube insertion (yes or no) (Fig. 3).

The RCT follows a blinded outcome assessment design; screening, enrolment, and randomization are conducted by unblinded researchers with knowledge of arm allocations, whilst all visits from post-randomization to study completion are managed by researchers blinded to arm allocations (Fig. 3). An unblinded statistician created and uploaded the randomization sequence into REDCap, the secure electronic research database. To safeguard blinding, staff receive training in blinding procedures, and access to sensitive data (e.g. randomization sequence files, REDCap randomization module) is restricted to designated unblinded staff only. Study materials are prepared in opaque envelopes, identifiable only to unblinded staff to mitigate unintentional unblinding. Should unintentional unblinding occur, future participant follow-up is reallocated to a different blinded researcher. Emergency unblinding is generally unnecessary, as blinded researchers can refer participant safety or other concerns directly to unblinded colleagues in real-time. The study has recruited sufficient personnel to ensure both blinded and unblinded researcher roles are adequately staffed at all times. Additionally, contingency plans are in place to preserve blinding integrity in the event of any staffing challenges.

### 4.9 Data collection and management

This RCT uses multiple diagnostic tools to assess ear health and function for outcome evaluation. The hearScope™ video-otoscopy device visualizes the external auditory canal and tympanic membrane, whilst tympanometry using the Interacoustics Titan Middle Ear Analyzer detects middle ear fluid causing conductive hearing loss [46,47]. Hearing sensitivity levels (pure-tone audiometry) are tested using the hearScreen® Smartphone Screening Audiometer [48], and the Interacoustics Titan Middle Ear Analyzer assesses inner ear health (cochlea outer hair cell function) via otoacoustic emissions to identify sensorineural hearing loss [49].

The OM-6 questionnaire evaluates health-related QoL in children with recurrent OM [50], and the PLUM and HATS are culturally appropriate questionnaires which aid in early detection of hearing and speech issues by gauging parent/carer perceptions of their child's hearing and speech [51,52]. A motivational outcomes questionnaire, developed using components of the Self Determination Theory Treatment Self-Regulation Questionnaire and the Perceived Competence Scale, measures the autonomous motivation of families to complete their assigned program, their perceived confidence and self-efficacy relating to program participation, and the extent to which its outcomes have met their expectations using a seven-point Likert scale [53].

In line with the RE-AIM framework, used in public health and implementation science to assess the impact and sustainability of health interventions, data to assess the implementation fidelity of the BBC intervention plus hand hygiene within the RCT will be collected using structured checklists [54]. These checklists include the assessment of the quality of intervention delivery at enrolment and quality of intervention performance at enrolment and post-randomization, along with a parent/carer checklist to confirm their level of understanding of the intervention at enrolment (Table 2) [54].

All research staff receive specialist training in study procedures (clinical and non-clinical) to ensure accuracy and reliability, along with International Council for Harmonization Good Clinical Practice (ICH-GCP) training to uphold standards in ethical conduct, data handling, and participant safety [55].

Data is collected using physical forms, securely stored at The Kids Research Institute Australia, and secure electronic devices store de-identified otoscopy, tympanometry, audiometry, and otoacoustic emissions data on HearX and Titan Suite servers. All data is transferred to Electronic Case Report Forms in the REDCap database, with accuracy of data entry ensured through double-checking and verification processes.

### 4.10 Primary and secondary statistical methods

A Bayesian logistic regression model will be used for the primary analysis on the binary endpoint $Y_{i}\in \left\{0,1\right\}$ for participant i=1,2,…,N. Currently, there are two arms and three recruitment pathways, however the model is designed to allow additional arms or recruitment pathways to be introduced in the future. A participant may be randomized to arm j=0,1,…,J (where j=0 is the control arm and j=1 is the intervention arm) and be recruited via pathway r=0,1,…,R (where r=0 is the PCH otolaryngology/audiology outpatient departments, r=1 is the PCH otolaryngology surgical pathway, r=2 is Djaalinj Waakinj and r≥3 for other approved sites). Let $x_{i}$ and $\omega_{i}$ be vectors of length J and R and contain the arm allocation and recruitment pathway, respectively, for participant i. Here, $x_{ij}=1$ if participant i is allocated to arm j and $x_{ij}=0$ otherwise. Similarly, $\omega_{ir}=1$ if participant i is recruited via recruitment pathway r and $\omega_{ir}=0$ otherwise. We use a Bernoulli model with a logistic link function below:

$$Y_{i}∼\text{Bernoulli}\left(\text{logi}t^{-1}\left(\pi_{i}\right)\right)$$

$$\pi_{i}=\alpha +\sum_{j=1}^{J}x_{ij}\beta_{j}+\sum_{r=1}^{R}\omega_{ir}\gamma_{r}$$

Here, $\pi_{i}$ is the log odds of OME resolution between 4 and 6 weeks post-randomization for participant i. The log odds for a participant allocated to the control arm and recruited via the PCH otolaryngology/audiology outpatient pathway is α, and $\beta_{j}$ and $\gamma_{r}$ are the adjusted log odds ratios of arm j compared to the control arm and recruitment pathway r compared to the PCH otolaryngology/audiology outpatient pathway, respectively. We assign the following weakly informative prior probability distributions to the model parameters:

$$\alpha ∼N\left(\text{logit}\left(0.17\right),0.3^{2}\right)$$

$$\beta_{j},\gamma_{r}∼N\left(0,1\right)\;\forall j=1,\ldots ,J,r=1,\ldots ,R$$

The posterior distribution of the quantity of interest ($\beta_{1}$) will be estimated from the primary analysis and will be used to inform trial adaptation decisions.

Statistical analyses for secondary outcome measures will involve using descriptive statistics to estimate rates of natural OME resolution from tympanometry results indicating OME persistence or resolution, analyzed using data collected during eligibility screening prior to enrolment and from control arm participants. Discharge rates from otolaryngology and audiology care will be tracked as percentages for both arms. Hearing outcomes will be compared using changes from baseline (week 0) in the four-frequency average hearing thresholds to those at weeks 4-to-6 and 24-to-32, analyzed using Bayesian linear models adjusted for baseline and recruitment site. QoL will be evaluated by analyzing changes in OM-6 outcomes using descriptive statistics, whilst PLUM and HATS outcomes will undergo additional analysis using a Bayesian proportional odds model. An economic analysis will compare costs of diagnosing and managing OME in the RCT as opposed to standard care, evaluating per-patient costs for OME diagnosis, tympanometry tube insertion, and cost benefits measured by OME resolution rates in AUD. High-throughput qPCR on the BioRad CFX96 platform will measure bacterial loads and otopathogen densities associated with OME *(Hemophilus influenzae, Streptococcus pneumoniae, Moraxella catarrhalis, Staphylococcus aureus, and Group A Streptococcus)* using validated qPCR primers, probes and reaction conditions [[56], [57], [58]]. The changes in bacterial densities over time between study arms will be ascertained through statistical analysis, assessing reductions or increases in bacterial load for each species of interest. Adverse events will be analyzed based on their number and nature in both trial arms. To align with the trial's flexible approach to study appointments, any results obtained outside of scheduled time points will be subject to sensitivity analyses to assess their impact on primary and secondary outcomes.

### 4.11 Interim analysis and decision criteria

The RCT will involve regular Bayesian updates to assess pre-planned decision criteria efficiently, ensuring timely answers to research questions [59]. The pre-planned adaptations include stopping recruitment for superiority or futility based on the primary analysis. The first interim analysis will occur after 100 eligible participants have completed the four-to-six-week post-randomization follow-up (referred to as ‘completers’), and subsequent updates for every 50 additional completers for the remainder of the RCT. Only data for eligible participants reaching weeks 4-to-6 will be used in the primary analysis.

Decision criteria for superiority and futility were identified using trial simulation to control for power and type one error. The BBC intervention plus hand hygiene will be declared superior at a pre-specified analysis, and recruitment into the trial may stop if the decision ruler for superiority is met:

$$P\left(\beta_{1}>\log\left(1.1\right)\right)>0.975$$

Alternatively, at a pre-specified analysis, the trial may stop recruitment for futility if the decision rule for futility is met:

$$P\left(\beta_{1}>\log\left(1.1\right)\right)<0.05$$

### 4.12 Clinical oversight

For participants recruited through Western Australian pediatric hospital pathways, a multidisciplinary team (MDT) structure will be utilized to review the clinical progress of each participant. The MDT consists of an otolargyngology Clinical Nurse Specialist, Consultant/Registrar, and Audiologist from each participating health service, as well as a study researcher, who collectively review clinical findings and formulate a care plan that aligns with the 2020 Otitis Media Guidelines [4], enabling alteration, escalation, or discharge from care to occur as clinically appropriate. Following the MDT review, care plans are delivered to parent(s)/carer(s) via phone call. For participants recruited through the Djaalinj Waakinj pathway, Research Audiologists provide ongoing clinical oversight to ensure care provided through the study aligns with the 2020 Otitis Media Guidelines [4].

### 4.13 Community consultation

In Australia, there is a prominent focus on research efforts dedicated to improving the health and wellbeing of Aboriginal people and communities in response to their experiences of ongoing health inequities. These inequities stem from the enduring impacts of colonization, including displacement, loss of cultural foundations, and systemic socioeconomic disadvantage [60,61]. Despite this focus, research involving Aboriginal people in Australia has a complex history of exploitation, misrepresentation, and marginalization [61]. In recent decades, approaches to research in the Aboriginal space have begun to shift from the treatment of Aboriginal people and communities as research subjects towards their inclusion as active partners and key stakeholders throughout the research process [43,44,61]. This shift is driven by researchers, organizations, and guidelines that acknowledge and understand the importance of adopting ethical, community-driven approaches that prioritize self-determination, foster genuine collaboration, and uphold cultural respect, resulting in research outputs that are both beneficial and culturally responsive [43,44,61].

The RCT is guided by an Aboriginal Community Advisory Group (ACAG), consisting of community members representing the south metropolitan area of Perth, Western Australia. The ACAG emphasized the importance of prioritizing research on childhood ear disease that focuses on reducing hospital visits, minimizing medication use, and avoiding surgical interventions. Recognizing the potential of the BBC plus hand hygiene to achieve these priorities, the ACAG endorsed conducting a RCT to test its efficacy. The research team provides regular updates to the ACAG about the progress of the RCT, incorporating their feedback to ensure the perspectives of Aboriginal children and families are upheld in all facets of research conduct. The ACAG's efforts highlight the critical role of culturally appropriate, community-driven health research in achieving meaningful outcomes for Aboriginal communities [43]. Given the diverse nature of communities across WA, in-depth community consultation with appropriate local community members and organizations will be conducted prior to adding any new WA study sites.

Additionally, The Kulunga Aboriginal Research Unit at The Kids Research Institute Australia continue to provide guidance, cultural expertise, and community navigation support to ensure this trial meets the standards for Aboriginal Health Research [62].

### 4.14 Data and participant safety monitoring

As rationalized by the NHMRC (2018) guidelines on Data Safety Monitoring Boards (DSMBs), a DSMB will not be convened as the BBC intervention plus hand hygiene and RCT procedures pose no significant risks to participants [63,64]. To maintain trial oversight, a Trial Advisory Group (TAG), comprising of key investigators, study staff, and an external advisor oversee the study, reviewing progress and addressing highlighted issues on a quarterly basis. An independent statistician will review the interim analyses data and share outcomes with the TAG for evaluation against decision criteria.

Safety data will be gathered at routine study visits through participant self-reporting and clinician/researcher observations. Any Adverse Events or Serious Adverse Events will be promptly reported following NHMRC guidelines to maintain participant safety and study integrity [64].

## 5 Discussion

Without cost-effective, non-invasive OME treatments to reduce tertiary care demand and wait times, many children will continue to face preventable hearing loss, delaying speech, learning, and social development, ultimately affecting academic achievement and employment opportunities [65,66]. Untreated OME increases demand for otolaryngology and audiology services and burdens families with costs for specialist care, devices (e.g. hearing aids), and lost workdays [23,67]. Low-cost and minimally invasive interventions like BBC plus hand hygiene could improve outcomes for children and reduce the impact of these burdens.

This is the first RCT to formally assess the efficacy of the BBC intervention plus hand hygiene, an essential step in expanding non-invasive OME treatment options for children aged two to seven years. Although BBC plus hand hygiene was designed and is currently implemented as a prevention program, establishing its treatment efficacy could lead to it becoming a standardized recommendation in clinical management guidelines for OME. For instance, the schedule of ear health screenings for Aboriginal and at-risk children within Western Australia offers multiple timepoints for introducing BBC plus hand hygiene as an OME treatment measure if proven effective [68]. Routine BBC plus hand hygiene implementation could decrease outpatient referrals, OME-related hospital admissions, and reduce the need for tympanostomy tube insertions by resolving OME in some children, potentially lowering costs for both health systems and families accessing out-of-pocket healthcare and preventing unnecessary exposure to surgical and anesthesia-related risks [14,25].

Non-invasive approaches, such as autoinflation devices that utilize positive pressure to clear middle ear fluid, have shown benefits in reducing OME persistence and improving OME-specific QoL [69]. However, the cost of these devices (e.g. Otovent® or EarPopper™) ranging from between $30-$240 AUD (approximately $20 – $200 USD) limits their accessibility, particularly in lower-income and developing settings [70]. Given the global prevalence of OME, especially in rural areas with limited specialist care [71], the BBC plus hand hygiene intervention offers a freely accessible alternative requiring no specialized equipment or training. As BBC is a free resource, evaluating its efficacy through an RCT aligns with global health goals stipulated in the World Health Organization's ‘World Report on Hearing’, which advocates for cost-effective, evidence-based interventions to reduce the global burden of ear disease [72].

Publishing the protocol for this adaptive RCT promotes transparency, reproducibility, and methodological rigor. Sharing it on a peer-reviewed platform ensures adaptive features and decision rules are documented accurately and clearly, reducing bias in outcome interpretation. Additionally, an open-access protocol supports researchers to replicate, build upon, or adapt the approach to a broader range of health issues.

## Additional notes

This manuscript was written based on the current version of the RCT protocol. Elements of the protocol may be subject to change throughout the course of the RCT due to the adaptive nature of its design.

## CRediT authorship contribution statement

**Jaimee R. Rich:** Writing – review & editing, Writing – original draft, Visualization, Validation, Project administration, Methodology, Investigation, Formal analysis, Data curation, Conceptualization. **Michael Dymock:** Writing – review & editing, Writing – original draft, Visualization, Validation, Supervision, Software, Resources, Methodology, Funding acquisition, Formal analysis, Data curation, Conceptualization. **Elke J. Seppanen:** Writing – review & editing, Writing – original draft, Visualization, Resources, Methodology, Formal analysis. **Elena Montgomery:** Writing – review & editing, Writing – original draft. **Tanisha Cayley:** Writing – review & editing, Project administration, Investigation, Data curation, Conceptualization. **Tamara Veselinović:** Writing – review & editing, Resources, Project administration, Investigation, Data curation, Conceptualization. **Greta Bernabei:** Writing – review & editing, Resources, Project administration, Investigation, Data curation. **Anri Lester:** Writing – review & editing, Project administration, Investigation, Data curation. **Amy Hannigan:** Writing – review & editing, Resources, Investigation. **Nicole Irvine:** Writing – review & editing, Resources, Investigation. **Kerryn Gidgup:** Writing – review & editing, Project administration, Investigation, Data curation. **Edna Ninyette:** Writing – review & editing, Project administration, Investigation, Data curation. **Steph Bray:** Writing – review & editing, Project administration, Investigation, Data curation. **Tu Trang Tran:** Writing – review & editing, Resources, Methodology, Investigation, Funding acquisition, Conceptualization. **Valerie M. Swift:** Writing – review & editing, Visualization, Validation, Supervision, Resources, Project administration, Methodology, Conceptualization. **Melinda Edmunds:** Writing – review & editing, Project administration, Methodology, Conceptualization. **Natalie Strobel:** Writing – review & editing, Methodology, Funding acquisition, Conceptualization. **Daniel McAullay:** Writing – review & editing, Methodology, Funding acquisition, Conceptualization. **Julie Marsh:** Writing – review & editing, Validation, Software, Resources, Methodology, Funding acquisition, Conceptualization. **Evelyn Tay:** Writing – review & editing, Validation, Software, Resources, Methodology. **Lea-Ann S. Kirkham:** Writing – review & editing, Visualization, Validation, Supervision, Resources, Formal analysis. **Ruth B. Thornton:** Writing – review & editing, Visualization, Resources, Formal analysis. **Robyn S.M. Choi:** Writing – review & editing, Writing – original draft, Visualization, Validation, Supervision, Methodology, Funding acquisition, Conceptualization. **Lydia Timms:** Writing – review & editing, Writing – original draft, Visualization, Validation, Supervision. **Emily Jackson:** Writing – review & editing, Writing – original draft, Visualization, Validation, Supervision. **Jafri Kuthubutheen:** Writing – review & editing, Visualization, Validation, Supervision, Resources, Methodology, Funding acquisition, Conceptualization. **Peter C. Richmond:** Writing – review & editing, Visualization, Validation, Supervision, Resources, Methodology, Funding acquisition, Conceptualization. **Christopher G. Brennan-Jones:** Writing – review & editing, Writing – original draft, Visualization, Validation, Supervision, Resources, Project administration, Methodology, Investigation, Funding acquisition, Formal analysis, Data curation, Conceptualization.

## Limitations

The public availability of the BBC plus hand hygiene intervention may result in control arm families accessing the program during the RCT, although its historically inconsistent implementation renders this unlikely [29]. Concurrent interventions may influence the outcomes of the BBC plus hand hygiene intervention; however, families are asked to discontinue any ear treatments that are not clinically prescribed during the for-to-six-week intervention period. Updated medical history and safety data is collected to monitor this, along with recording any use of essential treatments (e.g. antibiotics). Human factors may cause variations in intervention delivery despite standardized training. Consistence in program delivery will be achieved through ongoing training of research staff and clear protocols for intervention delivery. Level of parent(s)/carer(s) involvement may impact adherence to the routine; therefore, families partake in structured, interactive demonstrations by trained staff using step-by-step guides and checklists to promote consistency.

## Ethical considerations

This RCT follows the principles outlined in the Declaration of Helsinki, ensuring the ethical conduct of research and the protection of participants' rights, safety, and wellbeing [73]. This RCT is approved by the Child and Adolescent Health Service Human Research Ethics Committee (Approval No. RGS 5133), the Western Australian Aboriginal Health Ethics Committee (Approval No. HREC 1130), and the Curtin University Human Research Ethics Committee (Approval No. HRE2025-0072). Participants will receive ongoing follow-up care throughout and post-RCT through standard care pathways (e.g. through Western Australian pediatric hospitals and Djaalinj Waakinj). The BBC plus hand hygiene intervention will be offered post-RCT to control arm participants who wish to complete the BBC component. All participants will be fully informed about the study's objectives, methods, potential risks, benefits, and their rights, including their ability to withdraw without consequence. Informed consent is sought from the parent(s)/carer(s) of all participants.

## Declaration of generative AI and AI-assisted technologies in the writing process

No generative A.I. was used in the development of this manuscript.

## Funding

This trial and the Djaalinj Waakinj Centre for Ear and Hearing Health is funded by the Western Australian Department of Health Research Translation Project scheme, the Western Australian Future Health Research and Innovation Fund, the Stan Perron Charitable Foundation, and the National Health and Medical Research Council (GNT #2015750). JRR also gratefully acknowledges the ongoing support of The Kids Research Institute Australia Wesfarmers Centre of Vaccines and Infectious Diseases and the Research Training Program in the form of Higher Degree by Research stipend scholarships.

## Declaration of competing interest

The authors declare that they have no known competing financial interests or personal relationships that could have appeared to influence the work reported in this paper.

## Data Availability

No data was used for the research described in the article.
